# Awakening not associated with an increased rate of cortisol secretion

**DOI:** 10.1098/rspb.2024.1844

**Published:** 2025-01-15

**Authors:** Samantha Klaas, Thomas J. Upton, Eder Zavala, Michael Lawton, Sophie Bensing, Katarina Berinder, Ileana Botusan, Marianne Grytaas, Paal Methlie, Marianne Øksnes, Georgina Russell, Dimitra A. Vassiliadi, Stafford L. Lightman

**Affiliations:** ^1^ Laboratory for Integrative Neuroscience and Endocrinology, University of Bristol, Bristol BS1 3NY, UK; ^2^ Centre for Systems Modelling and Quantitative Biomedicine, Department of Metabolism and Systems Science, University of Birmingham, Birmingham B15 2TT, UK; ^3^ Population Health Sciences, Bristol Medical School, University of Bristol, Bristol BS8 2PN, UK; ^4^ Department of Endocrinology, Karolinska University Hospital, Stockholm 171 76, Sweden; ^5^ Department of Medicine, Haukeland University Hospital, Bergen 5020, Norway; ^6^ Department of Clinical Science, Faculty of Medicine, University of Bergen, Bergen 5020, Norway; ^7^ Department of Endocrinology, Evangelismos Hospital, Athens 106 76, Greece

**Keywords:** cortisol, cortisol awakening response, microdialysis, circadian rhythm, human, sleep

## Abstract

Cortisol is released upon activation of the hypothalamic–pituitary–adrenal axis, varies across the day, possesses an underlying diurnal rhythm and is responsive to stressors. The endogenous circadian peak of cortisol occurs in the morning, and increases in cortisol observed post-awakening have been named the cortisol awakening response (CAR) based on the belief that the act of waking up stimulates cortisol secretion. However, objective evidence that awakening induces cortisol secretion is limited. We used a mixed effects model with a linear spline fitted to the data to examine tissue-free cortisol measurements obtained from 201 healthy volunteers by automated ambulatory microdialysis before and after awakening in their home environments. We also examined rate of change of cortisol depending on sleep duration and relative timing. We found no evidence for a change in the rate of cortisol increase in the hour after waking when compared with the hour prior to waking. We instead observed substantial interindividual variability in the absolute concentration and rate of change of cortisol levels, and differences in dynamics that may be attributable to duration and relative timing of sleep. Based on these results, we strongly suggest caution is needed when interpreting cortisol measurements solely obtained in the hour after waking.

## 1. Introduction

Cortisol is released upon activation of the hypothalamic–pituitary–adrenal (HPA) axis to meet both anticipated and unexpected demands of cognitive, metabolic, cardiovascular and immunological systems [1]. It achieves this through the combination of a circadian rhythm that prepares the body in anticipation of the day ahead, and the ability to rapidly respond to acute stressors. Cortisol provides energy by modulating glucose production, releasing fatty acids from storage and promoting protein catabolism [2]. It is also both anti- and pro-inflammatory, regulating immune activity in response to pathogens, and vasoactive—regulating blood flow to muscles and the brain [3].

Based on the assumption that wakening is a ‘stressor’ that induces cortisol secretion, dynamic changes observable soon after the time of wakening have come to be defined as the ‘cortisol awakening response’, or CAR [4]. Almost exclusively measured in saliva [4], it is claimed that the CAR is a distinct phenomenon superimposed upon the circadian rhythm of cortisol [5,6] and an ancient adaptive feature preparing the body in anticipation of the energy needs for the day ahead [7,8]

The CAR has attracted a lot of interest and is now a widely accepted term in the field of psychoneuroendocrinology. It is cited as a reliable measure of adrenocortical activity [9], which has led to its common use as a biomarker of stress reactivity and HPA functionality [10,11]. Compared with healthy controls, altered CAR patterns are reported for Cushing’s [12], chronic fatigue [13], depression and psychosis [14,15], post-traumatic stress disorder [16] and obesity [17]. However, several studies have made comment on high interindividual variation in the CAR [14,18]. Furthermore, a recent study of 140 healthy participants comparing five different tests of HPA activity showed no obvious physiological correlates of the CAR, and the authors questioned the biological meaning of this measure [19].

One crucial limitation of the CAR is reliance on saliva measurement, which—aside from recognized methodological issues related to accurate timing while awake [4]—does not permit assessment of the rate of change of cortisol prior to wakening, that is, while still asleep. Therefore, to date, the only method for truly assessing changes in cortisol specifically related to the act of waking has been in a laboratory setting using total cortisol concentrations measured in blood samples. While laboratory protocols might be beneficial for their ability to impose specific controls and establish mechanistic connections, they are neither feasible nor practical as a mass-adoptable method for ambulatory waking-related cortisol assessment, where pre-waking cortisol measurements are needed. We attempted to address this by instead assessing free cortisol before and after waking in interstitial fluid samples in an ambulatory setting [20]. The method, based on the well-established technique of microdialysis [21], permits automatic sample collection during daily activity and sleep [22] and correlates well with serum [22,23] and plasma [24] cortisol. A recent analysis of 24 h cortisol profiles in 214 healthy volunteers sampled at home and self-selecting sleep and wake times confirmed the daily rhythmicity of cortisol in tissue [24], with peak concentrations occurring soon after waking up. However, that analysis only considered cortisol in relation to clock time rather than wake time and did not consider differences in cortisol change that might be specifically related to waking itself.

Therefore, in this current work, we specifically investigated changes of tissue-free cortisol collected before and after waking in a cohort of healthy volunteers who were sampled at home to determine whether we could separate any effect of waking from the rhythmic daily rise of cortisol that peaks in the morning.

## 2. Methods

### (a) Study design

The aim of this analysis was to investigate whether waking was associated with an increase in tissue-free cortisol in a cohort of night-sleeping healthy volunteers, free from active medical diagnoses or regular medication, and who chose their own sleep period. To do this, we analysed data collected as part of the Dynamic Hormone Diagnostics (ULTRADIAN) observational cohort study (NCT02934399) and available in a public repository (UiB Open Research Data, https://doi.org/10.18710/5TW8YF).

The methods of data collection and preparation are described in detail in Upton *et al*. [24] but briefly, involved the use of a portable microdialysis-based sampling system that enabled continuous sampling in subcutaneous tissue. Each participant completed a 24 h sampling session generating 72 individual samples, representing a 20 min sampling resolution. During the sampling period participants continued normal activities, and the sleep period occurred in the participant’s own home. Participants were free to select their own sleep and wake times, which were self-reported in an activity diary for both the night prior to and night of sampling. Concentrations of free cortisol and other adrenal steroids were quantified in each sample using liquid chromatography–tandem mass spectrometry.

Participants were recruited between 2016 and 2020 at study sites in Bergen (Norway, Regional Ethics Committee West, 2015/872), Bristol (UK, South West—Frenchay Research Ethics Committee IRAS, 181429), Athens (Greece, Evangelismos Hospital, 208/20 October 2015) and Stockholm (Sweden, Swedish Ethical Review Authority, 2016/1463−31/4).

### (b) Study participants

We assessed data from *n* = 214 male and female participants aged 18–68 who had met eligibility criteria to be included in the ULTRADIAN healthy volunteer cohort, as previously described [24]. For this work, we excluded 13 participants who either did not have a documented wake time recorded (*n* = 10) during the sampling period, or where there were more than 2 missing data points within the time window 60 min before and after waking (*n* = 3).

### (c) Statistical methods

Preparation, visualization and statistical feature analysis of the time-series data were done in Python (3.9.16) using the libraries NumPy, SciPy and Pandas and the plotting libraries Seaborn and Matplotlib, while regression models were implemented in R (4.3.1) using the libraries lspline and nlme.

To investigate changes in cortisol concentration in relation to wake time, and to account for individual variation in hormone trajectories over time, we used a mixed effects regression model with a linear spline fitted to the data.

Mixed models estimate a population average trajectory along with individual specific trajectories. The random effects provide an estimate of the variability between the population average and individual specific trajectories. The spline knot was set at the time *t*_waking_ = 0 to permit comparison of the rate of cortisol change (reflected by the slope of the curve) per minute before and after waking. The dependent variable was concentration of cortisol, with each study participant as the random effect. We estimated random effects for the intercept and for both the slopes before and after the knot point with an unstructured covariance matrix. The model formula for cortisol was thus


lme(Cortisol∼lspline(SampleTime,knots=c(0),marginal=TRUE),random=∼lspline(SampleTime,knots=c(0),marginal=TRUE)ǀParticipantID)

Standardized parameters were obtained by fitting the model on a standardized version of the dataset. 95% Confidence Intervals (CIs) and *p*-values were computed using a Wald *t*-distribution approximation. For all statistical tests, the threshold for statistical significance was defined as *p *< 0.05.

In the dataset, each microdialysis measurement was timestamped as the midpoint of the cumulative sample collected over a 20 min period (for example, a sample collected between 06.00 and 06.20 would be labelled 06.10) and then the time of each sample was realigned relative to individual wake time. Since wake time did not necessarily coincide exactly with this midpoint sample time, we included any samples collected within the period of 70 min before and after wake time. Furthermore, recognizing that inclusion of samples spanning wake time (that is, individual samples where collection began before waking and ended after waking) could potentially bias the measurement in that time window if there was a change in slope, we conducted a sensitivity analysis comparing results of the model with and without these samples (electronic supplementary material, table S1). The resulting estimates were similar and confidence intervals in the model without waketime samples (focussing on the fixed effects) overlapped with the model including all data, so the model containing all data was used to describe the results.

To investigate differences in the rate of change of cortisol with respect to waking and sleep duration, the time-series data were resampled to a 1 min frequency using cubic spline interpolation. In the case of missing data, the maximum time permitted for interpolation was 20 min. Next, the first-order derivative of the resulting hormone time series was calculated providing the slope at each and every time point (providing the instantaneous rate of change in cortisol concentration per unit of time). While the rate of change provided useful visual information, it was not used as an input for the regression models; therefore, the 1 min interpolation did not impact the statistical analysis.

Sleep duration was calculated by subtracting sleep offset from onset times. For each participant, cortisol secretion start time was defined as the time at which hormone concentration reached 20% of the increase from nadir concentration (lowest cortisol level within 7 h prior to waking) to morning peak (maximum cortisol level in the period 4 h prior to 2 h post waking), consistent with the definitions used previously [24].

## 3. Results

### (a) No effect of waking on rate change of morning-free tissue cortisol

**Figure 1.**
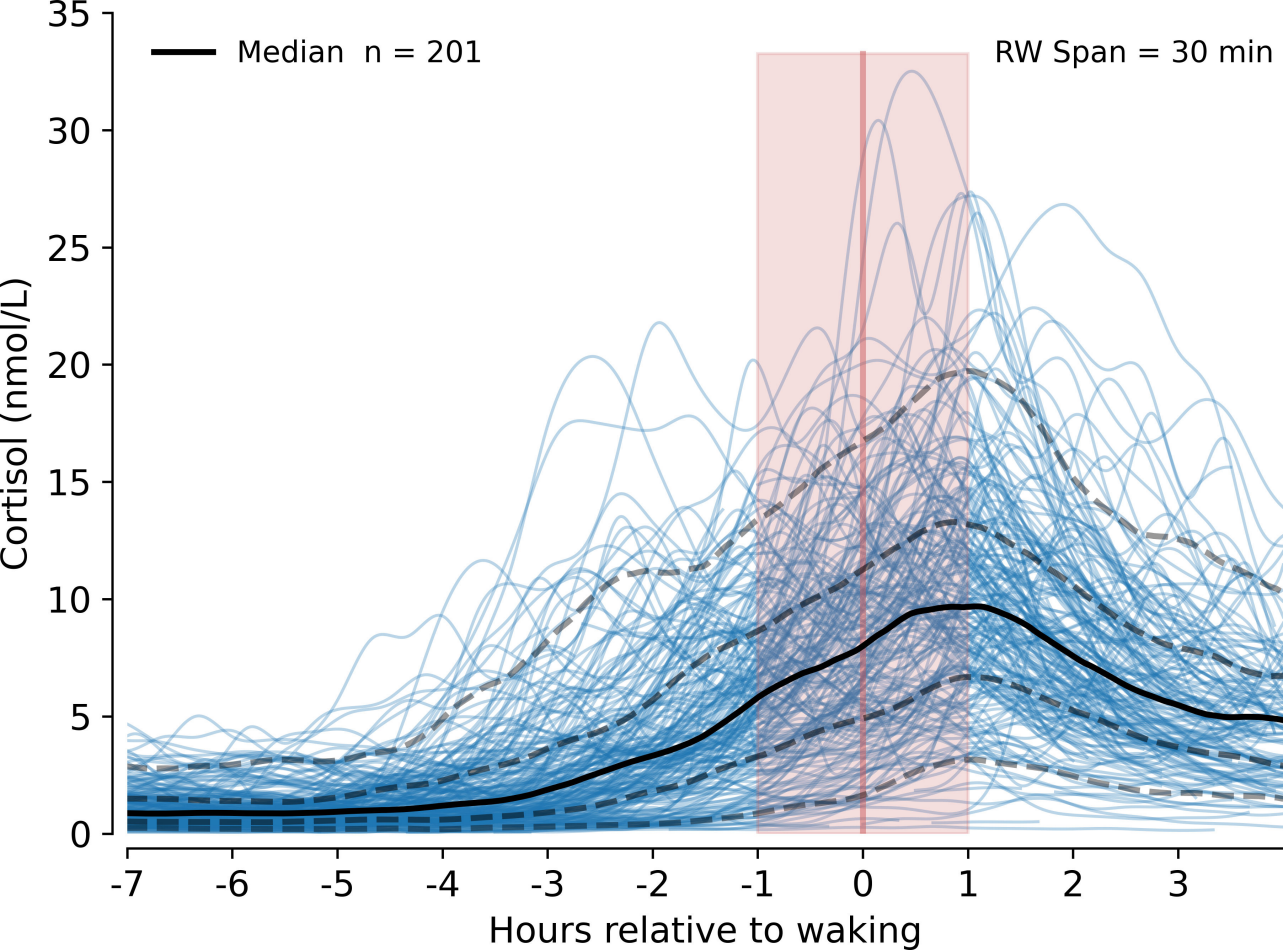
Overnight tissue-free cortisol profiles in *n* = 201 healthy volunteers aligned by wake time. The rolling median (solid black) and quartile range (dashed black) lines (5th, 25th, 75th and 95th, bottom to top) are shown against the background of individual participant data (blue lines). Vertical line represents time of waking; shaded area represents 1 hr pre- and post-waking time. RW = rolling window.

We examined sequential measurements of cortisol in the hour immediately before and then after waking in 201 ambulatory healthy participants to determine whether there was a difference in the rate of change that could be attributed to the act of waking (table 1, figure 1).

**Table 1.** Description of an ambulatory cohort of healthy volunteers (*n* = 201 total, *n* = 197 for sleep time and duration).

	*n* (%), then mean ± s.d.
females / males	107 (53%) / 94 (47%)
BMI (kg m^2^)	23.5 ± 2.76
age (years)	42.9 ± 14.2
sleep time	23.27 ± 0.58
wake time	07.02 ± 1.15
sleep duration	456 ± 72 min
cortisol onset, clock time	04.13 ± 1.22
cortisol peak, clock time	07.45 ± 1.21
cortisol onset, relative to waking	−187 ± 89.0 min
cortisol peak, relative to waking	+36 ± 59.5 min

For each participant, the mean number of samples included for analysis was 7.1 ± 0.57 (range 5–8). While we found very strong evidence that cortisol increased with time in the hour prior to waking (rate of change 0.039 nmol L^−1^ min^−1^, 95% CI [0.030, 0.048], *p *< 0.001), we found no evidence for a difference in cortisol change per minute in the hour after waking compared with the hour prior to waking (−0.008 nmol L^−1^ min^−1^, 95% CI [−0.022, 0.006], *p* = 0.280). Within the cohort, however, we found evidence of substantial between-participant variability. The variation in waking cortisol (considering the random effect) between individual specific values and the population mean (8.58 nmol L^–1^, 95% CI [7.88, 9.27], *p *< 0.001) was large (s.d. 4.94 nmol L^–1^). Further, individual cortisol changes per minute prior to waking (considering the random effect) also varied substantially in relation to the mean (s.d. 0.062 nmol L^−1^ min^–1^), implying that cortisol changed much more rapidly in some participants than in others—where there was either no increase or a negative slope prior to waking. There was also individual variation (considering the random effect) in the difference in cortisol change per minute in the hour after waking in relation to the mean (s.d. = 0.096 nmol L^−1^ min^–1^).

We found a positive correlation between the random intercepts and slopes during the pre-wake period (*r* = 0.63), suggesting that participants with a larger increase in cortisol per minute prior to waking were more likely to have higher waking cortisol values, while a negative correlation (*r* = −0.61) in the post-wake hour observations implies that for higher waking cortisol participants, the change in cortisol per minute between the pre-wake and post-wake periods was likely to be smaller. There was a negative correlation (*r* = −0.73) between the two random slopes, which suggests that individuals whose pre-wake rate of cortisol change is higher tend to have a lower difference in slope post-waking.

In addition to cortisol, other adrenal hormones rise in response to adrenocorticotrophic hormone (ACTH), also with peak levels around the time of waking. Recent analysis indicates that one such metabolite, plasma 18-hydroxycortisol, is not only very highly correlated with plasma cortisol but is also better correlated with tissue 18-hydroxycortisol than plasma cortisol is with tissue cortisol [24]. Therefore, to validate our result for cortisol, we repeated the analysis substituting cortisol with 18-hydroxycortisol in the same cohort of participants using the same methodology (electronic supplementary material, figure S1). Like cortisol, we found very strong evidence that 18-hydroxycortisol increased with time (1.12 pmol L^−1^ min^−1^, 95% CI [0.72, 1.52], *p *< 0.001) but no evidence for a change following waking (−0.43 pmol L^−1^ min^−1^ , 95% CI [−1.07, 0.22], *p* = 0.20).

### (b) Cortisol peak occurs close to wake time

We examined the relationship of the nadir and peak cortisol concentrations in relation to wake times and found that on average, peak values occurred just under 40 min after waking having risen from a nadir 3 h prior to waking (table 1, figure 1).

**Figure 2.**
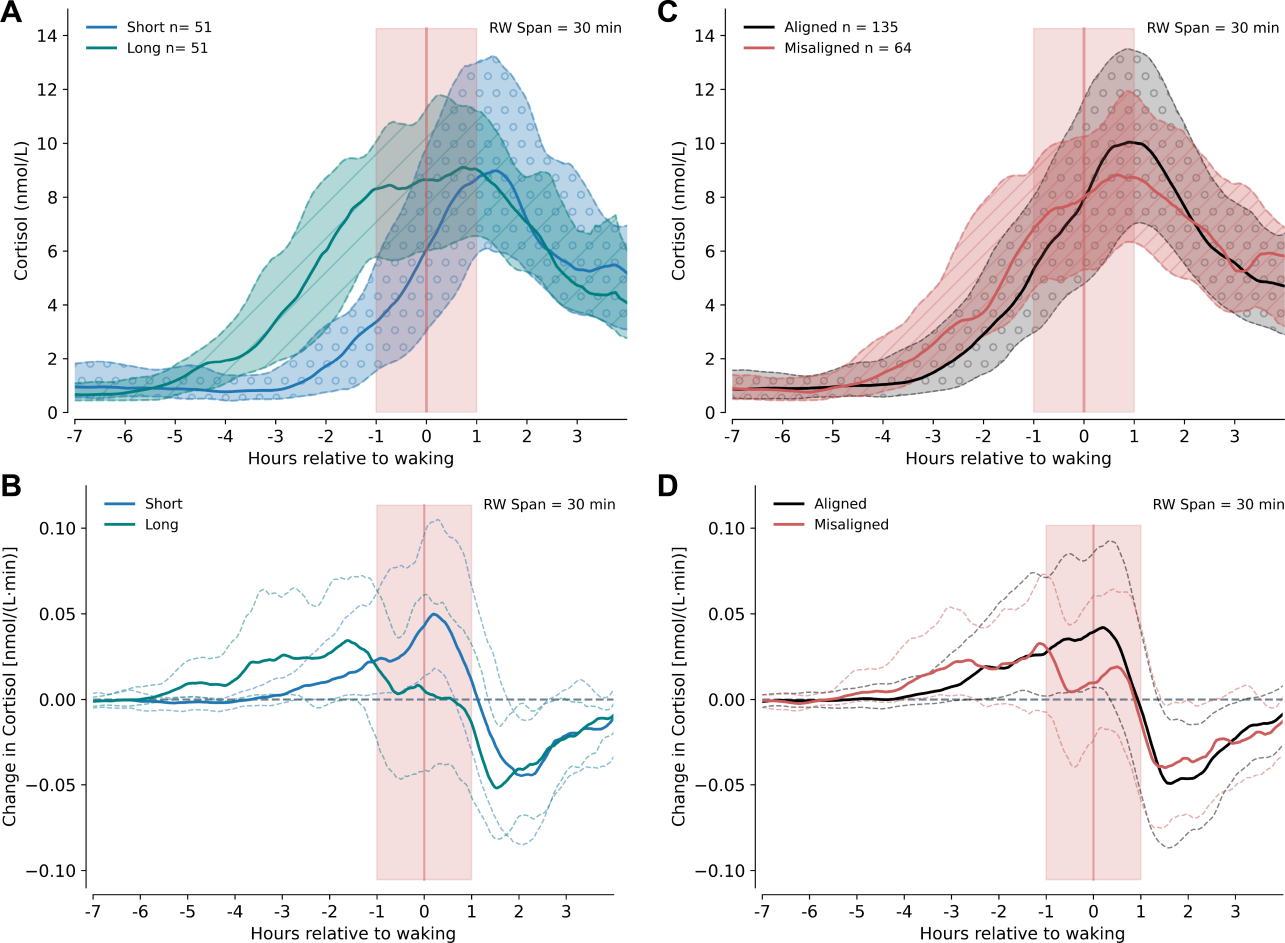
Cortisol values and cortisol rate of change in different sleep conditions. (A) Rolling median values for short (*n* = 51, solid blue) and long (*n* = 51, solid green) sleepers and (B) in aligned (*n* = 135, solid black) and misaligned (*n* = 64, solid red) sleepers. Rate of change of cortisol [nmol/( L. min)] in relation to wake time for short and long sleep (C) and misaligned versus aligned sleep (D) conditions. The 25th–75th centiles are shown with dotted lines and in hatched areas. RW = rolling window.

We again noted the range of interindividual variation in the cohort. While peak cortisol concentrations occurred in most participants following waking (*n* = 162, 81%, mean 59.8 ± 32.5 min), in a substantial minority the peak occurred in the 60–120 min post-waking period (*n* = 77, 38%, mean 87.4 ± 20.5 min) and in a smaller group cortisol peaked prior to waking (*n* = 39, 19%, mean −61 ± 45 min), implying that cortisol values were declining after waking in those participants.

### (c) Sleep timing and duration may influence the rate of change of cortisol around waking

We next considered differences in sleep duration or whether consistency of sleep timing might impact cortisol change around wake time. We investigated differences in the slopes and the rate of change of cortisol secretion in participants with short versus long sleep times, and in participants with a wake time inconsistent with the previous night's sleep.

To investigate the potential effect of sleep duration, the cohort was split into participants with short (≤25th centile, *n* = 51, mean 369 ± 34 min), and long (≥75th centile, *n* = 51, mean 548 ± 34 min) durations (figure 2*a*). In the short sleep group, cortisol increased prior to waking (0.052 nmol L^−1^ min^−1^ , 95% CI [0.033, 0.071], *p *< 0.001), a similar rate to the whole cohort, without any evidence of a change in the slope of increase in the hour following waking (−0.002 nmol L^−1^ min^−1^, 95% CI [−0.030, 0.025], *p* = 0.863). However, in the long sleep group we found on average no cortisol increase in either the hour prior to (0.009 nmol L^−1^ min^−1^, 95% CI [−0.006, 0.025], *p* = 0.239) or following wake (−0.002 nmol L^−1^ min^−1^, 95% CI [−0.027, 0.023], *p* = 0.867). In the long sleep group, the median maximal rate of cortisol increase occurred 97 min before waking, compared with 12 min after waking in the short sleeping group (figure 2*b*).

To investigate the potential effect of wake time variation, the whole cohort was split into participants whose wake time on the sampling day differed from the wake time on the previous morning by 60 min or more (misaligned cohort, *n* = 64, mean offset 103.5 ± 47 min) and the remainder where wake time was within 60 min of the previous morning (aligned cohort, *n* = 135, mean offset 17 ± 16 min, figure 2*c*).

In both aligned and misaligned sleepers, we found evidence that cortisol was increasing in the hour before wake (aligned group average rate: 0.049 nmol L^−1^ min^−1^, 95% CI [0.038, 0.061], *p *< 0.001; misaligned group average rate: 0.017 nmol L^−1^ min^−1^, 95% CI [0.002, 0.031], *p* = 0.02). When compared with the pre-waking slope, we did not find evidence for a change in the rate of cortisol change per minute in the hour after waking for either aligned (average post-wake rate −0.015 nmol L^−1^ min^−1^, 95% CI [−0.033, 0.004], *p* = 0.114) or misaligned (average post-wake rate 0.006 nmol L^−1^ min^−1^, 95% CI [−0.017, 0.030], *p* = 0.610) groups (figure 2*c*, table 2). However, in the aligned group, the median maximum rate of cortisol increase occurred at +12 min after waking, compared with 68 min before waking in the misaligned cohort (figure 2*d*).

**Table 2.** Changes in cortisol with respect to wake time in healthy volunteers. Results of a mixed effects regression model with a linear spline fitted to the data used to estimate rate of cortisol change (95% CI) before and after waking and to predict mean cortisol and standard deviation (s.d.) at wake time. Total number of observations included = 1447.

condition	cortisol change prior to wake [nmol L^−1^ min^−1^]	cortisol change after wake [nmol L^−1^ min^−1^]	***p*** **‐value**	population mean cortisol (nmol L^−1^) at wake ± s.d.
all *n* = 201	0.039 [0.030–0.048]	−0.008 [−0.022–0.006]	0.280	8.58 ± 4.94
short sleep *n* = 51	0.052 [0.033–0.071]	−0.002 [−0.030–0.025]	0.863	7.03 ± 5.53
long sleep *n* = 51	0.009 [−0.006–0.025]	−0.002 [−0.027–0.023]	0.867	8.97 ± 4.05
aligned *n* = 135	0.049 [0.038–0.061]	−0.015 [−0.033–0.004]	0.114	8.72 ± 5.27
misaligned *n* = 64	0.017 [0.002–0.031]	0.006 [−0.017–0.030]	0.609	8.16 ± 4.25

## 4. Discussion

In this analysis, we confirmed that on average, free-tissue cortisol levels, as measured using an ambulatory microdialysis technique in healthy volunteers, increase before waking and peak shortly afterward. Using a mixed effects regression model with a linear spline at wake time, we were unable to find evidence for a difference in the rate of change of cortisol in the hour before and after waking. Instead, we noted large interindividual variability in both absolute levels and rate of cortisol change and further found that sleep duration and relative timing were also potential influencers of changes in cortisol around waketime. These are important findings that must be considered carefully given the very large number of studies already in publication that make conclusions based on cortisol measurements solely obtained after waking, using methodologies that assume that waking itself provokes a ‘cortisol awakening response’ [4].

Consensus guidelines exist that provide methodological standards for cortisol measurement after waking based on serial post-wake salivary assessment [4]. Although this methodology can describe the data that have been obtained, it does so based on the assumption that the rate of cortisol change must have altered as a result of waking, since it is impossible to observe the pre-waking state. Therefore, considering our current finding, it is worth considering the evidence that supports this assumption.

While there are numerous examples of studies demonstrating cortisol increase in the period after waking (for example [9,25], which are commonly cited to support the existence of a CAR), we found that there are very few studies that specifically examine pre- and post-waking cortisol. In 1992, Späth-Schwalbe *et al*. [25] measured cortisol before and after awakening in a small cohort (14 healthy men, aged 20−34). While the study was done in laboratory conditions, participants adhered to their own typical wake up time. The authors concluded that spontaneous awakening led to brief cortisol increases, and that the sleep–wake transition is responsible for this. However, quantitative analyses showing increased secretion immediately after awakening are missing from the paper, and the pre- and post-awakening time window used to make such a conclusion was not specified. In fact, the authors’ own observation was that cortisol decreased from spontaneous awakening to 11.00 in the morning and they concluded that activation of the HPA axis in the morning is terminated soon after awakening.

In 1999, Born and colleagues [26] assessed ACTH and cortisol in 15 healthy young people (mean age 25.2) with a regular but unspecified sleep–wake rhythm, over 3 nights. This study exposed them to either forced early awakening with advance warning, surprise early waking (both at 06.00, presumably before habitual wake time), or waking later at 09.00 (long sleep, presumably at or after habitual wake time). In the ‘short surprise’ condition, an increase in ACTH in the hour before waking was noted, although this was not accompanied by an increase in cortisol. Absolute change in cortisol, measured from immediately pre- to an unspecified period after waking, was only different in the ‘surprise’ forced awakening group, increasing by a similar amount in both the ‘short’ and ‘long’ sleeping groups. Overall, while this study may support the existence of ‘wake anticipation’, and that early, ‘surprise’ (forced) awakening may be a stressor, it does not provide evidence that waking in general results in increased cortisol secretion.

In 2007, Wilhelm *et al*. [18] noted upon review of the literature that despite its common use, there was still no conclusive evidence supporting the existence of the CAR. In an attempt to address this, blood sampling of cortisol was done between 21.00 and 08.00 in a cohort of 16 male students, and the authors reported a steeper post-awakening rise compared with pre-awakening cortisol, leading to the conclusion that awakening induces cortisol secretion. However, it should be noted that this result was again observed when participants were forcibly awakened. Indeed, the one subject who woke up naturally before the scheduled time was excluded from the data analysis. As noted, forced awakening itself can be stressful, depending on whether the time is representative of an individual’s habitual waking time, and thus whether their sleep was disrupted prematurely [27]. Not only does time of awakening influence cortisol changes post-awakening [28], but in this case the external manipulation may itself account for the increased cortisol secretion. Further, only 75% of the participants had a significant measurable increase in cortisol after waking during home sampling, compared with 100% in the laboratory, which implies changes in the cortisol response on the day of their pre- and post-awakening sampling schedule.

Our findings are consistent with the premise that the most substantial determinant of cortisol change around waking is the timing of the measurement in relation to the endogenous cortisol rhythm. This is concordant with at least three prior studies looking at cortisol responses at different times of the circadian cycle [29–31]. Dettenborn *et al*. [29] found that forced awakenings during the night did not lead to significant cortisol increases, whereas forced awakenings in early morning hours did, which is consistent with cortisol differences being related to phase of the daily rhythm rather than a direct response to awakening. Federenko *et al*. [30] studied 31 students and found no mean cortisol increase after waking up from a nap taken between 18.45 and 20.30, a time of day during which cortisol is typically falling. Furthermore, in a multiday in-laboratory protocol of varying sleep schedules, cortisol rose after waking in healthy individuals during a circadian phase corresponding to 3.40−3.45 but not during the circadian phases corresponding to the afternoon [31]. Such conclusions support the inference that post-wake cortisol increases will be observed when samples are obtained at a time of circadian increase in cortisol and not during the circadian decline. Early waking time is commonly considered a covariate influencing post-wake salivary cortisol measures [4]. For example, Bakusic *et al*. [32] noted in assessment of 18 participants that earlier waking time was associated with higher post-wake saliva cortisol values—a likely explanation being that those participants who wake early are waking during a period of the cortisol rhythm associated with more rapid increase compared with those who wake later. In summary, these findings, and the coincident time of waking with the peak of cortisol suggest that the morning rise in the hormone, which has an intrinsic circadian rhythm, anticipates habitual wake time. However, this conclusion does not preclude the possibility that other factors, including sleep itself, might also have a modulatory effect on pre-wake changes in cortisol. To test this robustly will require further studies that consider individuals' typical sleep patterns, wake time and sufficient sampling resolution to detect cortisol change.

Further, we found large inter-individual variation in morning cortisol dynamics—not only in amplitude, but also differences in cortisol slopes and peak times depending on sleep duration and relative wake time. We observed in our data that while, for some individuals, cortisol continued to increase from awakening to a post-wake peak, in others the peak had already occurred before waking, or no change in cortisol was observed. This is not surprising given that small changes in the time of awakening—as well as differences related to sex, light influx, weekday versus weekend sampling, stress levels, genetics [5] and potentially the expected time of wake [26]—may influence the measurement of cortisol at any given time. A high degree of variation is also seen in post-waking cortisol measurements in saliva [33], including in closely controlled laboratory settings [34]. High day-to-day variability in post-wake salivary cortisol may also be observed within the same individual [32]. A meta-analysis of 301 participants who provided saliva samples at various waking times also concluded that a high degree of interindividual variability exists in the magnitude of what was termed diurnal cortisol cycles, additionally noting that approximately 10%–20% of the classified healthy volunteers did not show diurnal variation, describing theses as ‘flat cycles’ [35]. An important caveat for this study is that any wake time samples were actually excluded from analysis, owing to *a priori* assumptions that the CAR must exist. Taken together, our study provides further evidence of the complexity of measuring such a dynamic and variable hormone as cortisol close to the circadian maxima. Therefore, although convenient, a great deal of caution must be exercised when attempting to interpret and extrapolate the biological meaning of a few measurements solely obtained after waking.

We acknowledge that our study has some limitations. We analysed an existing dataset from a study whose original purpose was not specifically to test hypotheses related to waking cortisol. In the study, wake time was self-reported. Although self-report is generally well associated with objective measures of wake, at least in healthy people [36], use of an objective measure of sleep timing, quality and especially wake time would be highly desirable for any future studies in this area. When combined with computational mathematical analysis [37] such an approach could provide considerable further insight into the physiology of awakening. A further potential criticism is that the microdialysis system that we used captured sample cumulatively over a 20 min period and it therefore might not be able to detect very subtle changes in cortisol. The counterargument to this is that in some cases, the slope of post-wake cortisol did increase, and this was detectable; however, overall, such cases did not represent the general finding, which was simply of a continued increase in cortisol that terminates soon after waking. Finally, the validity of tissue-free cortisol measurements might be questioned as it is a relatively novel method compared with assessment in saliva or blood. While there is evidence that tissue-free cortisol may lag behind total plasma measurements [24], a cross-check with 18-hydroxycortisol, which has a smaller lag, yielded the same result.

In conclusion, in our examination of 201 healthy volunteers sampled in an ambulatory home setting, we found that the rate of cortisol change did not increase as a consequence of waking, a result that is more supportive of cortisol anticipation and the influence of intrinsic rhythmicity than reaction to waking. Within the cohort, we observed a high degree of interindividual variation that could at least partly be attributed to differences in sleep and differences in endogenous phase. When conducting research on cortisol change around wake time, it is crucial that future studies carefully consider the hypothalamic pituitary axis in addition to sleep and behaviour.

## Data Availability

Data and code used for the primary analyses are available in an online public repository Dryad [38]. Supplementary material is available online [39].

## References

[1] Lightman S, Terry JR. 2014 The importance of dynamic signalling for endocrine regulation and drug development: relevance for glucocorticoid hormones. Lancet Diabetes Endocrinol. **2**, 593–599. (10.1016/S2213-8587(13)70182-7) 24731665

[2] Oster H, Challet E, Ott V, Arvat E, de Kloet ER, Dijk DJ, Lightman S, Vgontzas A, Van Cauter E. 2017 The functional and clinical significance of the 24-hour rhythm of circulating glucocorticoids. Endocr. Rev. **38**, 3–45. (10.1210/er.2015-1080) 27749086 PMC5563520

[3] Russell GM, Kalafatakis K, Lightman SL. 2015 The importance of biological oscillators for hypothalamic-pituitary-adrenal activity and tissue glucocorticoid response: coordinating stress and neurobehavioural adaptation. J. Neuroendocrinol. **27**, 378–388. (10.1111/jne.12247) 25494867 PMC4539599

[4] Stalder T *et al*. 2022 Evaluation and update of the expert consensus guidelines for the assessment of the cortisol awakening response (CAR). Psychoneuroendocrinology **146**, 105946. (10.1016/j.psyneuen.2022.105946) 36252387

[5] Clow A, Thorn L, Evans P, Hucklebridge F. 2004 The awakening cortisol response: methodological issues and significance. Stress **7**, 29–37. (10.1080/10253890410001667205) 15204030

[6] Vargas I, Lopez-Duran N. 2020 The cortisol awakening response after sleep deprivation: is the cortisol awakening response a ‘response’ to awakening or a circadian process? J. Health Psychol. **25**, 900–912. (10.1177/1359105317738323) 29076400

[7] Contreras CM, Gutierrez-Garcia AG. 2018 Cortisol awakening response: an ancient adaptive feature. J. Psychiatry Psychiatric Disord. **2**, 29–40. (10.26502/jppd.2572-519X0038)

[8] Schulz P, Kirschbaum C, Prüßner J, Hellhammer D. 1998 Increased free cortisol secretion after awakening in chronically stressed individuals due to work overload. Stress Med. **14**, 91–97. (10.1002/(SICI)1099-1700(199804)14:2<91::AID-SMI765>3.0.CO;2-S)

[9] Pruessner JC, Wolf OT, Hellhammer DH, Buske-Kirschbaum A, von Auer K, Jobst S, Kaspers F, Kirschbaum C. 1997 Free cortisol levels after awakening: a reliable biological marker for the assessment of adrenocortical activity. Life Sci. **61**, 2539–2549. (10.1016/s0024-3205(97)01008-4) 9416776

[10] Anderson T, Wideman L. 2017 Exercise and the cortisol awakening response: a systematic review. Sports Med. Open **3**, 37. (10.1186/s40798-017-0102-3) 29019089 PMC5635140

[11] Law R, Clow A. 2020 Stress, the cortisol awakening response and cognitive function. Int. Rev. Neurobiol. **150**, 187–217. (10.1016/bs.irn.2020.01.001) 32204832

[12] Roa SLR, Elias PCL, Castro M, Moreira AC. 2013 The cortisol awakening response is blunted in patients with active Cushing’s disease. Eur. J. Endocrinol. **168**, 657–664. (10.1530/EJE-12-0982) 23392212

[13] Roberts ADL, Wessely S, Chalder T, Papadopoulos A, Cleare AJ. 2004 Salivary cortisol response to awakening in chronic fatigue syndrome. Br. J. Psychiatry **184**, 136–141. (10.1192/bjp.184.2.136) 14754825

[14] Dedovic K, Ngiam J. 2015 The cortisol awakening response and major depression: examining the evidence. Neuropsychiatr. Dis. Treat. **11**, 1181–1189. (10.2147/NDT.S62289) 25999722 PMC4437603

[15] Mondelli V *et al*. 2010 Abnormal cortisol levels during the day and cortisol awakening response in first-episode psychosis: the role of stress and of antipsychotic treatment. Schizophr. Res. **116**, 234–242. (10.1016/j.schres.2009.08.013) 19751968 PMC3513410

[16] Rauch SAM, King A, Kim HM, Powell C, Rajaram N, Venners M, Simon NM, Hamner M, Liberzon I. 2020 Cortisol awakening response in PTSD treatment: predictor or mechanism of change. Psychoneuroendocrinology **118**, 104714. (10.1016/j.psyneuen.2020.104714) 32446108 PMC7984524

[17] Wallerius S, Rosmond R, Ljung T, Holm G, Björntorp P. 2003 Rise in morning saliva cortisol is associated with abdominal obesity in men: a preliminary report. J. Endocrinol. Invest. **26**, 616–619. (10.1007/BF03347017) 14594110

[18] Wilhelm I, Born J, Kudielka BM, Schlotz W, Wüst S. 2007 Is the cortisol awakening rise a response to awakening? Psychoneuroendocrinology **32**, 358–366. (10.1016/j.psyneuen.2007.01.008) 17408865

[19] Abelson JL, Sánchez BN, Mayer SE, Briggs H, Liberzon I, Rajaram N. 2023 Do diurnal salivary cortisol curves carry meaningful information about the regulatory biology of the HPA axis in healthy humans? Psychoneuroendocrinology **150**, 106031. (10.1016/j.psyneuen.2023.106031) 36801587 PMC12082605

[20] Bhake RC, Leendertz JA, Linthorst ACE, Lightman SL. 2013 Automated 24-hours sampling of subcutaneous tissue free cortisol in humans. J. Med. Eng. Technol. **37**, 180–184. (10.3109/03091902.2013.773096) 23547774

[21] Plock N, Kloft C. 2005 Microdialysis—theoretical background and recent implementation in applied life-sciences. Eur. J. Pharm. Sci. **25**, 1–24. (10.1016/j.ejps.2005.01.017) 15854796

[22] Bhake R, Russell GM, Kershaw Y, Stevens K, Zaccardi F, Warburton VEC, Linthorst ACE, Lightman SL. 2020 Continuous free cortisol profiles in healthy men. J. Clin. Endocrinol. Metab. **105**, e1749–e1761. (10.1210/clinem/dgz002) 31529059

[23] Bhake RC, Kluckner V, Stassen H, Russell GM, Leendertz J, Stevens K, Linthorst ACE, Lightman SL. 2019 Continuous free cortisol profiles—circadian rhythms in healthy men. J. Clin. Endocrinol. Metab. **104**, 5935–5947. (10.1210/jc.2019-00449) 31355884

[24] Upton TJ *et al*. 2023 High-resolution daily profiles of tissue adrenal steroids by portable automated collection. Sci. Transl. Med. **15**, eadg8464. (10.1126/scitranslmed.adg8464) 37343084

[25] Späth-Schwalbe E, Schöller T, Kern W, Fehm HL, Born J. 1992 Nocturnal adrenocorticotropin and cortisol secretion depends on sleep duration and decreases in association with spontaneous awakening in the morning. J. Clin. Endocrinol. Metab. **75**, 1431–1435. (10.1210/jcem.75.6.1334495) 1334495

[26] Born J, Hansen K, Marshall L, Mölle M, Fehm HL. 1999 Timing the end of nocturnal sleep. Nature **397**, 29–30. (10.1038/16166) 9892349

[27] Späth-Schwalbe E, Gofferje M, Kern W, Born J, Fehm HL. 1991 Sleep disruption alters nocturnal ACTH and cortisol secretory patterns. Biol. Psychiatry **29**, 575–584. (10.1016/0006-3223(91)90093-2) 1647222

[28] Edwards S, Evans P, Hucklebridge F, Clow A. 2001 Association between time of awakening and diurnal cortisol secretory activity. Psychoneuroendocrinology **26**, 613–622. (10.1016/s0306-4530(01)00015-4) 11403981

[29] Dettenborn L, Rosenloecher F, Kirschbaum C. 2007 No effects of repeated forced wakings during three consecutive nights on morning cortisol awakening responses (CAR): a preliminary study. Psychoneuroendocrinology **32**, 915–921. (10.1016/j.psyneuen.2007.06.011) 17681429

[30] Federenko I, Wüst S, Hellhammer DH, Dechoux R, Kumsta R, Kirschbaum C. 2004 Free cortisol awakening responses are influenced by awakening time. Psychoneuroendocrinology **29**, 174–184. (10.1016/s0306-4530(03)00021-0) 14604599

[31] Bowles NP *et al*. 2022 The circadian system modulates the cortisol awakening response in humans. Front. Neurosci. **16**, 995452. (10.3389/fnins.2022.995452) 36408390 PMC9669756

[32] Bakusic J, De Nys S, Creta M, Godderis L, Duca RC. 2019 Study of temporal variability of salivary cortisol and cortisone by LC-MS/MS using a new atmospheric pressure ionization source. Sci. Rep. **9**, 1–12. (10.1038/s41598-019-55571-3) 31848390 PMC6917784

[33] Ross KM, Murphy MLM, Adam EK, Chen E, Miller GE. 2014 How stable are diurnal cortisol activity indices in healthy individuals? Evidence from three multi-wave studies. Psychoneuroendocrinology **39**, 184–193. (10.1016/j.psyneuen.2013.09.016) 24119668 PMC3869640

[34] Elder GJ, Ellis JG, Barclay NL, Wetherell MA. 2016 Assessing the daily stability of the cortisol awakening response in a controlled environment. BMC Psychol. **4**, 3. (10.1186/s40359-016-0107-6) 26818772 PMC4730747

[35] Stone AA, Schwartz JE, Smyth J, Kirschbaum C, Cohen S, Hellhammer D, Grossman S. 2001 Individual differences in the diurnal cycle of salivary free cortisol: a replication of flattened cycles for some individuals. Psychoneuroendocrinology **26**, 295–306. (10.1016/s0306-4530(00)00057-3) 11166492

[36] Sadeh A. 2011 The role and validity of actigraphy in sleep medicine: an update. Sleep Med. Rev. **15**, 259–267. (10.1016/j.smrv.2010.10.001) 21237680

[37] Grant AD, Upton TJ, Terry JR, Smarr BL, Zavala E. 2022 Analysis of wearable time series data in endocrine and metabolic research. Curr. Opin. Endocrin. Metab. Res. **25**, 100380. (10.1016/j.coemr.2022.100380) PMC9823090 36632470

[38] Klass S *et al*. 2024 Data for: Interstitial cortisol measurements aligned by wake time, healthy volunteers. Dryad Digital Repository. (10.5061/dryad.2280gb62w)

[39] Klaas S, Upton T, Zavala E, Lawton M, Bensing S, Berinder K *et al*. 2024 Supplementary material from: Awakening not associated with an increased rate of cortisol secretion. Figshare (10.6084/m9.figshare.c.7595624) PMC11732391 39809311

